# Analysis of the 24-h biting patterns and human exposures to malaria vectors in south-eastern Tanzania

**DOI:** 10.1186/s13071-024-06521-0

**Published:** 2024-10-30

**Authors:** Muwonge C. Mukisa, Jibsam J. Kassano, Yohana A. Mwalugelo, Charles Ntege, Najat F. Kahamba, Marceline F. Finda, Betwel J. Msugupakulya, Halfan S. Ngowo, Fredros O. Okumu

**Affiliations:** 1 Environmental Health and Ecological Sciences Department, Ifakara Health Institute, P. O. Box 53, Ifakara, Tanzania; 2 School of Life Science and Bio-Engineering, The Nelson Mandela African Institution of Science and Technology, P.O. Box 447, Arusha, Tanzania; 3 National Malaria Control Division, Ministry of Health, P.O. Box 7272, Kampala, Uganda; 4 Department of Biomedical Science, Jaramogi Oginga Odinga University of Science and Technology, Bando, Kenya; 5 Department of Animal Biology and Conservation Science, School of African Regional Postgraduate Programme in Insect Science (ARPPIS), University of Ghana, Accra, Ghana; 6 School of Biodiversity, One Health, and Veterinary Medicine, University of Glasgow, Glasgow, UK; 7 Department of Vector Biology, Liverpool School of Tropical Medicine, Liverpool, UK; 8 Faculty of Health Science, School of Public Health, University of the Witwatersrand, Johannesburg, South Africa

**Keywords:** Persistent malaria transmission, Human behavior, Day-biting *Anopheles* mosquitoes, ITNs, Ifakara

## Abstract

**Background:**

Afrotropical malaria vectors are generally believed to bite nocturnally, leading to the predominant use of insecticide-treated nets (ITNs), which target indoor, nighttime-biting mosquitoes. This focus is reinforced by biases in entomological surveys, which largely overlook daytime mosquito activity. However, recent evidence challenges this paradigm, showing that *Anopheles* biting can extend way into the daytime, coinciding with human activities at dawn, daytime and evenings, suggesting a broader risk spectrum and potential protection gaps. We have therefore investigated the diurnal and nocturnal biting patterns of the malaria vectors *Anopheles arabiensis* and *Anopheles funestus* in south-eastern Tanzania, to better understand the scope of residual transmission and inform strategies for improved control.

**Methods:**

Host-seeking mosquitoes were collected hourly using miniaturized double net traps, both indoors and outdoors over 24-h periods between June 2023 and February 2024. Concurrently, human activities indoors and outdoors were monitored half-hourly to correlate with mosquito collections. A structured questionnaire was used to assess household members’ knowledge, perceptions and experiences regarding exposure to mosquito bites during both nighttime and daytime.

**Results:**

Nocturnal biting by *An. arabiensis* peaked between 7 p.m. and 11 p.m. while that of *An. funestus* peaked later, between 1 a.m. and 3 a.m. Daytime biting accounted for 15.03% of *An. arabiensis* catches, with peaks around 7–11 a.m. and after 4 p.m., and for 14.15% of *An. funestus* catches, peaking around mid-mornings, from 10 a.m. to 12 p.m. Nighttime exposure to *An*. *arabiensis* was greater outdoors (54.5%), while daytime exposure was greater indoors (80.4%). For *An. funestus*, higher exposure was observed indoors, both at nighttime (57.1%) and daytime (69%). *Plasmodium falciparum* sporozoites were detected in both day-biting and night-biting *An. arabiensis.* Common daytime activities potentially exposing residents during peak biting hours included household chores, eating, sleeping (including due to sickness), resting in the shade or under verandas and playing (children). From evenings onwards, exposures coincided with resting, socializing before bedtime and playtime (children). Nearly all survey respondents (95.6%) reported experiencing daytime mosquito bites, but only 28% believed malaria was transmissible diurnally.

**Conclusions:**

This study updates our understanding of malaria vector biting patterns in south-eastern Tanzania, revealing considerable additional risk in the mornings, daytime and evenings. Consequently, there may be more gaps in the protection provided by ITNs, which primarily target nocturnal mosquitoes, than previously thought. Complementary strategies are needed to holistically suppress vectors regardless of biting patterns (e.g. using larval source management) and to extend personal protection limits (e.g. using repellents). Additionally, community engagement and education on mosquito activity and protective measures could significantly reduce malaria transmission risk.

**Graphical Abstract:**

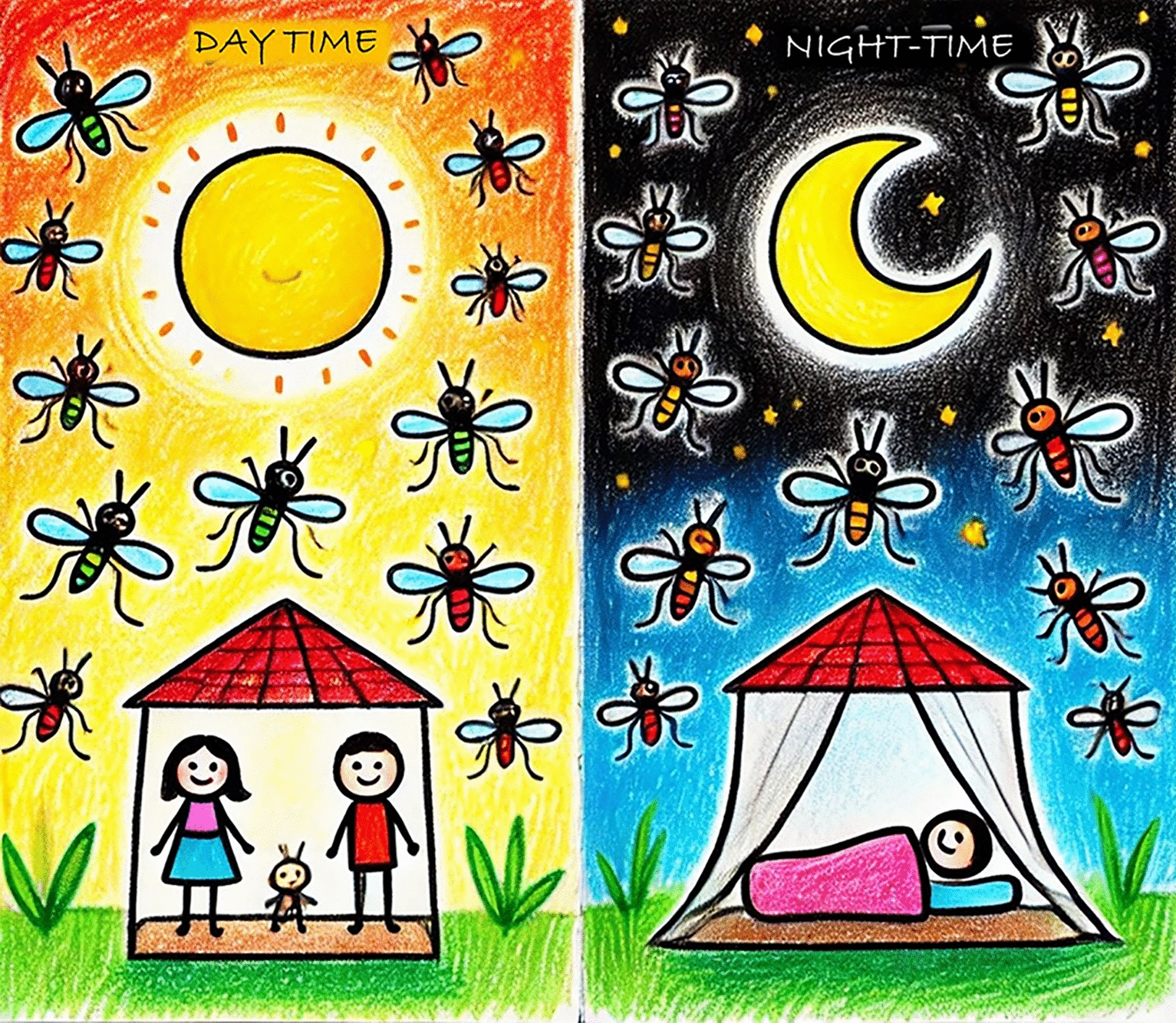

## Background

Despite significant advances in malaria eradication and elimination, malaria is still a substantial public health threat globally, with approximately 249 million cases and over 608,000 associated deaths in 2022 [1]. The main prevention strategies, insecticide-treated nets (ITNs) and indoor residual spraying (IRS) have significantly reduced malaria incidence, together contributing to nearly 80% of the progress made in Africa against the disease, according to a 2015 publication [2]. Similarly, in Tanzania, the adoption of ITNs and IRS, augmented with effective case management, has achieved notable reductions in malaria prevalence over recent years, cutting prevalence from 14% in 2016 to 8% in 2022 [3, 4]. These developments have inspired the prospects for potential elimination across many endemic regions, including those where transmission has been historically intense.

ITNs and IRS primarily target the nocturnal and indoor-biting behaviors of the major Afrotropical malaria vectors, *Anopheles gambiae *sensu lato (*An. gambiae *s.l.) and *Anopheles funestus*, effectively transforming human dwellings into lethal traps that reduce malaria transmission [5]. While these interventions primarily address indoor behaviors, their mosquitocidal attributes can accrue significant community-level benefits even for non-users [6–8], underscoring their critical role in tailored vector control strategies for public health management. Moreover, in certain circumstances, these tools can, to a certain degree, also impact vector populations that bite outdoors [9–11].

Despite significant advances in vector control, the path to malaria elimination faces numerous biological and behavioral challenges. The main threats include insecticide resistance, which diminishes the effectiveness of ITNs and IRS [12, 13], and drug resistance, particularly concerning artemisinin-based therapies crucial for treating malaria [14–16]. Additionally, mutations like the deletion of the *Plasmodium falciparum* HRP2 gene complicate malaria diagnosis by making parasites undetectable by standard rapid tests [17, 18]. The invasion and spread of the Asian malaria vector, *Anopheles stephensi*, particularly in urban eastern African settings, introduces a new dynamic, as this vector is less affected by traditional control measures [19–22]. In many settings, these challenges are further complicated by behavioral adaptations among vectors, such as increased outdoor feeding and biting during the daytime and in the early evenings and mornings when people are not under ITNs, allowing malaria transmission to persist despite high intervention coverage [23–27]. In one study in Bangui, Central African Republic, approximately 20–30% of daily exposure to indoor *Anopheles* biting happened during the day, suggesting a significant protection gap in such settings [23]. These changes underscore the need for dynamic vector management strategies that adapt to evolving vector behaviors to maintain the effectiveness of malaria interventions.

Unlike ITNs, which are mostly targeted at nocturnal biting behaviors of the main vectors like *An. gambiae* s.l. and *An. funestus*, IRS targets resting mosquitoes and larval source management targets mosquitoes at the source (mosquito breeding sites) [28–30]. Therefore, these two strategies can, to a large degree, function irrespective of the peak mosquito biting activity times [31], and Larval source management in particular can remain effective despite biological threats like insecticide resistance and outdoor biting [31, 32]. Unfortunately, for most people in sub-Saharan Africa, their defense against malaria is derived primarily from the protection offered by ITNs during nighttime when these vectors are most active. Moreover, the evidence is increasing of daytime biting by Africa’s main malaria vector species, coinciding with periods when people are engaged in activities such as farming and fetching water and thus not protected by ITNs [23, 33–37]. This behavior, whether innate or emergent in response to current interventions, significantly broadens the risk spectrum, further expanding the already substantial gaps in protection by strategies designed primarily for nocturnal intervention.

This evidence underscores a critical oversight in current vector control strategies, necessitating a re-evaluation and possible expansion of intervention focus to encompass vector activities during the daytime. Such adjustments are essential not only for safeguarding at-risk populations throughout their active hours but also for reducing the residual transmission of malaria. The profound implication of these studies is that ongoing malaria control efforts might be compromised by previously underappreciated vector behaviors, highlighting the need for a nuanced and flexible approach to both the surveillance and control strategies to accommodate the full behavioral spectrum of malaria vectors. In places where the main malaria vectors include *An. funestus*, especially in eastern and southern Africa [38], the increasing evidence of daytime and early morning biting behaviors can be particularly concerning. While IRS, which has historically been common in this region, may continue to be effective against these behaviors [31], its deployment is increasingly declining, in part because of logistical challenges, housing modifications and diminishing community acceptance, all of which can lead to upsurges in malaria cases [39–41].

The majority of field studies on malaria vectors have concentrated on the nighttime activities of the vectors, in line with the operational hours of ITNs. This predominant focus has led to a significant gap in current understanding of vector behaviors during the daytime, including early mornings and early evenings, times when human activity and human-mosquito interactions can be high but protection is low. This methodological bias has skewed the understanding of vector behavior and limited the effectiveness of interventions designed with a nocturnal bias. For these reasons, there is a pressing need for comprehensive studies that include 24-h behavioral assessments of vectors, which will inform the development of responsive and effective malaria control measures across the vector’s entire activity spectrum. Additionally, residents in malaria-endemic areas generally have diverse behaviors and housing structures that influence exposure to mosquito bites [25, 42–44], potentially compromising the effectiveness of ITNs and IRS. Despite the high coverage of these interventions, persistent malaria transmission in places such as rural southeastern Tanzania [45] underscores the necessity for detailed studies on vector behaviors beyond traditional monitoring hours. Understanding these local dynamics is essential for designing interventions that effectively curb malaria transmission, aligning with the elimination goals of many endemic countries.

This study therefore aimed to investigate the diurnal and nocturnal biting patterns of two major vectors, *Anopheles arabiensis* and *An. funestus*, in rural southeastern Tanzania—in an area characterized by high ITN usage but persistent moderate to high malaria prevalence [46]. The study also examined human activities and behaviors that could influence malaria vector biting risk both inside and outside homes. By analyzing these 24-h patterns of exposure, we aimed to better elucidate the gaps in control of the persistent malaria transmission in the area and to inform improved strategies for control and elimination.

## Methods

### Study area

**Fig. 1:**
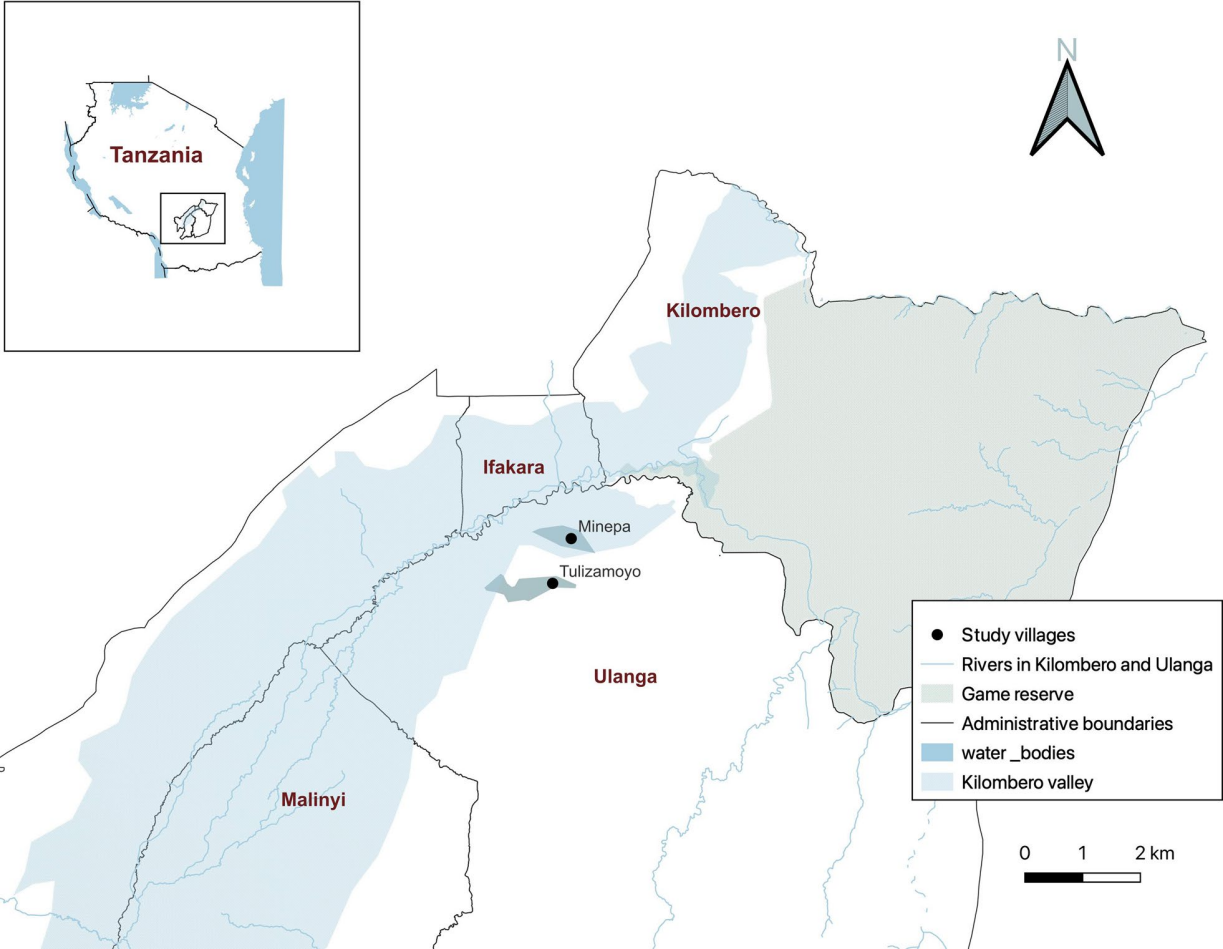
Map of the study area showing villages in Kilombero Valley where the study was conducted

The study was conducted in two rural villages, Tulizamoyo and Minepa, in Ulanga District which is within the Kilombero Valley in southeastern Tanzania, from June 2023 to February 2024 (Fig. 1). This valley experiences moderate to high malaria transmission, with prevalence rates varying from < 1% in semi-urban areas to > 50% in rural ones [46]. Malaria prevention in the area predominantly involves the use of ITNs, distributed primarily through the National Malaria Control Program via mass distribution campaigns and also through supplementary channels, such as antenatal visits and school net distribution programs. Currently, > 77% of households have at least one ITN [4]. Houses in these villages are typically constructed of mud or brick walls, occasionally plastered with concrete, and are roofed with either grass thatch or metal. The main economic activity is small-scale rice farming, sometimes supported by irrigation. The climatic conditions, with annual rainfall ranging from 1200 to 2100 mm and temperatures fluctuating between 23 °C and 27 °C [47], can support moderate to high mosquito densities year-round, and also continuous malaria transmission. *Anopheles funestus* and *An. arabiensis* are the main malaria vectors, the former being responsible for > 80% of malaria transmission, even where the latter is the more abundant [45, 48–50].

### Study design

This study involved concurrent entomological data collection and human behavior observations (HBOs) both indoors and outdoors over 24-h periods in the study area. Additionally, community perceptions and knowledge about day-biting mosquitoes and the associated risk of diseases were assessed through a structured questionnaire in a community survey. In each village, 12 sentinel houses reflecting local architectural styles were randomly chosen for weekly routine entomological surveillance and human behavior surveys. Then, 16 adult males were recruited following written informed consent from each village and trained in mosquito collection methods, including the use of the miniaturized double net (DN-Mini) technique [10] to participate in the study. The houses were sampled in rounds of four every 2 days with breaks to allow rest periods for volunteers, and time for the scientists and technicians to process the samples.

### Sampling and processing of adult mosquitoes

**Fig. 2:**
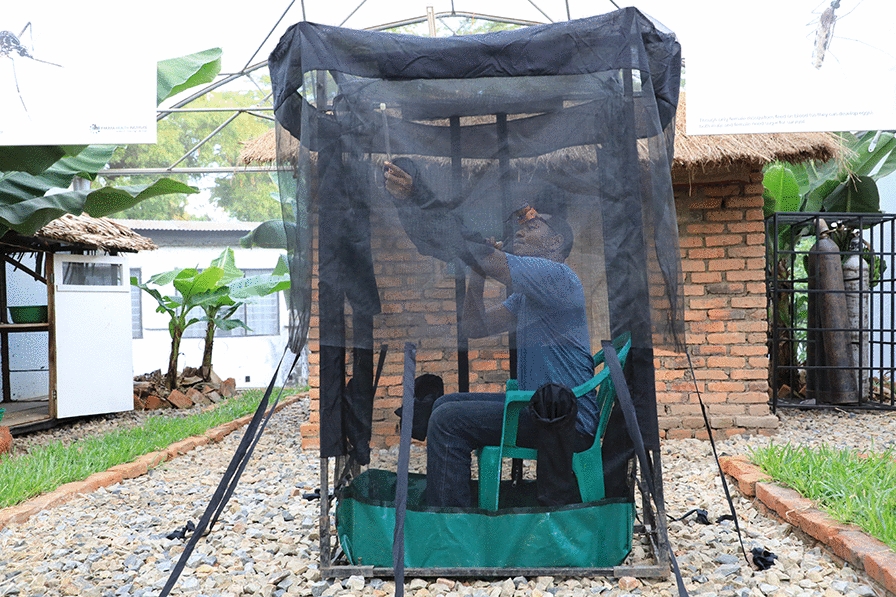
Miniaturized double net trap (DN-Mini) for sampling host-seeking mosquitoes indoors and outdoors. Picture was adapted from Limwagu et al. [10]

Host-seeking mosquitoes were collected both indoors and outdoors using a DN-Mini trap during 32 nights in each village (Fig. 2). The DN-Mini is an exposure-free sampling method that allows for direct estimation of the mosquito biting risk and can be used indoors or outdoors [10, 51]. The indoor DN-Mini trap was placed in the living room while the outdoor trap was placed in areas shaded by trees, with a radius of 5–10 m distant from the sentinel house. Mosquito collections were done hourly for 24 h, starting from 7 p.m. to 7 a.m. the following morning, by teams of four trained volunteers working in 6-h shifts to minimize fatigue and reduce collection bias. Each volunteer, stationed either inside or outside the target house, used a mouth aspirator to collect mosquitoes from the outer chambers of the DN-Mini every hour starting the 50th minute.

The collected mosquitoes were separated and classified by the hours of collection. Each morning, the mosquitoes were identified based on their morphological characteristics using taxonomic keys [52]. They were sorted by species, location and physiological status (unfed, fed, semi-gravid and gravid). Non-gravid females of *An. arabiensis* and *An. funestus* sensu lato (*An. funestus* s.l.) were dissected to assess physiological age by assessing parity, following the Detinova method [53]. Samples were then stored in labeled micro-centrifuge tubes for further analysis. The pooled hourly samples were analyzed by PCR assays and enzyme-linked immunosorbent assays (ELISA) for sibling species identification [54, 55] and detection of *Plasmodium* circumsporozoite proteins [56, 57], thereby providing insights into malaria transmission dynamics.

### Observations of the behaviors and activities of household members in the peri-domestic area

In addition to the entomological surveys, human activities and movements were observed directly to enable the quantification of exposures to mosquito bites in relation to human activities within the household vicinity. Data on human behaviors and activities in the peri-domestic area were collected half-hourly alongside mosquito sampling at the same houses to assess human exposure to mosquito bites over 24-h periods, both indoors and outdoors. Behavioral data were collected in a subset of the selected sentinel houses for entomological surveys in each village using a standardized observation form, pre-outlined with common activities, as previously described [25]. The houses were selected based on the presence of a willing qualified observer who was able to read and write. The observers, who were either consenting household members or trained relatives, tallied all the observed activities hourly, noting the age and sex of individuals involved. Each observer continuously tracked different activities conducted by household members from 7 a.m. in the morning, throughout the day and until all members had retired to bed at night. At that point, the observations stopped and were only resumed the following morning as soon as the first household member rose to begin daily activities.

### Assessment of knowledge, perceptions and experiences of community members regarding the risk of mosquito bites outside periods when ITNs are used

To understand community awareness regarding mosquito bites and associated disease risks outside of ITN protection hours, including mornings, daytime and evenings, a structured questionnaire survey was conducted that targeted household representatives in the same study villages. The survey, conducted in the Swahili language, examined the awareness and knowledge of community members as well as their daily activities that could expose them to mosquito bites. This information was used to construct a typical day for household members, correlating their activities with the observed 24-h mosquito biting patterns to enhance understanding of malaria transmission risk. A systematic random sampling approach was used to select households for participation. Kobo Toolbox software [58] was used to administer the survey via electronic tables, facilitated by trained interviewers. The survey was conducted between December 2023 and February 2024. A list of all households was obtained from the village leaders, and 91 households were randomly selected per village.

A total of 182 households participated in the survey. This sample size was estimated using a formula for determining a single population proportion [59], i.e. $n= \frac{{Z}^{2}*p (1-p)}{{d}^{2}}$, where $n$ was the sample size, $z$ was the statistic value at a 95% confidence level (1.96), $d$ was the level of precision (0.05) and *p*, the proportion of malaria prevalence in children in the Morogoro region (0.06) [4]. This calculation provided an initial sample size of approximately 87 households. Since the survey was conducted in two villages, this number was doubled to 174 households. Additionally, to account for a potential 5% non-response rate, an additional nine households were added to the survey, bringing the total to 182 households. In each village, sampling began by randomly selecting the first household and then continued systematically using a predetermined sampling interval.

### Data analysis

Data were initially entered into Microsoft Excel (Microsoft Corp., Redmond, WA, USA)and then imported into R statistical software version 4.3.1 for further processing and analysis [60]. A generalized linear mixed model (GLMM) fitted with a Bayesian approach was used [61] to model the hourly abundance of mosquitoes per person. The model incorporated time of collection and location as predictor variables, and mosquito counts as the response variable. Household identification (ID) and day of collection were included as random effects to account for variability between days and households. Mosquito counts were modeled using Poisson distributions with a log-link function. Each mosquito species was modeled separately. To attain convergence, each model species was run for 10^4^ iterations with 1000 burn-in periods. Model diagnostics included visual inspection of trace plots and evaluation using Gelman–Rubin statistics to assess convergence. Graphical representations were produced using the *ggplot2* package [62]. Correlation between human behavior and mosquito biting was done using descriptive statistics and visualization was done using figures [25, 63]. The results were expressed as relative risks with 95% credible intervals (CI).

Additionally, descriptive analyses were used to assess household members’ understanding and perceptions of the risk of mosquito bites and malaria transmission throughout the day, particularly during the daytime. Continuous variables were expressed as means and categorical variables were expressed as percentages.

## Results

### Mosquito catches indoors and outdoors during nighttime and daytime collections

**Fig. 3:**
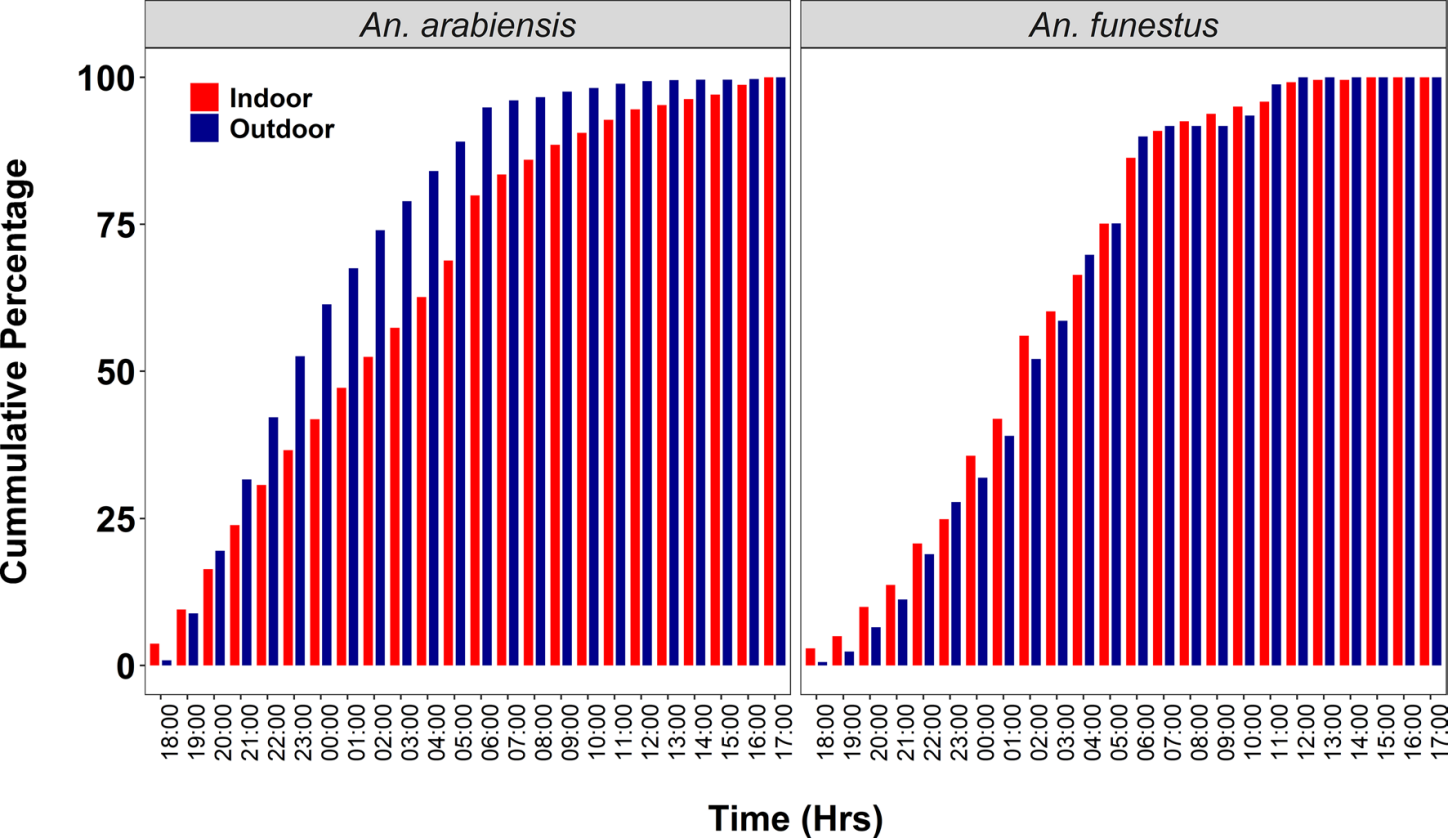
Cumulative percentages of *Anopheles funestus* and *Anopheles arabiensis* indoors and outdoors over the 24-h collection period

A total of 10,987 female *Anopheles* mosquitoes and 23,367 female culicine mosquitoes (*Culex pipiens* and *Mansonia uniformis*) were collected (Table 1). Of all female *Anopheles* mosquitoes collected during the study, the majority were caught at night, from 7 p.m. to 6 a.m. (85.2%), with only 14.8% caught during the day (6 a.m. to 7 p.m.). The cumulative percentages of the catches are shown in Fig. 3. During the nighttime collections, 45.6% of all *Anopheles* mosquitoes were caught indoors and 54.4% were caught outdoors (Table 1). However, during the daytime, 79.3% of all *Anopheles* collected were caught indoors, and 20.7% were caught outdoors. Culicine mosquitoes were also mainly caught at night (77.8%), with 48.5% of these caught indoors and 51.5% caught outdoors. During the daytime, 75.1% of Culicine were caught indoors and 24.9% were caught outdoors. Most of the *Anopheles* mosquitoes collected were *An. arabiensis* (10,283; 93.6%) and *An. funestus* (410; 3.7%); the other *Anopheles* species caught included *An. coustani* (232; 2.1%), *An. squamosus* (52; 0.5%) and *An. pharoensis*. On the other hand, the non-*Anopheles* mosquitoes caught comprised 19,785 (85%) *Culex quinquefasciatus* and 3582 (15%) *Mansonia uniformis*. In line with the objectives of the study, further statistical analysis focused on the two predominant malaria vectors, *An. arabiensis* and *An. funestus.*

**Table 1:** Diversity and densities of mosquitoes collected during the survey, using human-baited double net traps placed indoors or outdoors during daytime and nighttime

Mosquito catch	Nighttime mosquito collections			Daytime mosquito collections			Totals (Night & day catches)
	Indoors,* n* (%)	Outdoors,* n* (%)	Total catch (indoor and outdoor catches) (*n*)	Indoors,* n* (%)	Outdoors,* n* (%)	Total (indoor and outdoor catches)	
*Anopheles species*							
*An. arabiensis*	3976 (45.5%)	4761 (54.5%)	8737	1243 (80.4%)	303 (19.6%)	1546	10,283
*An. funestus*	201 (57.1%)	151 (42.9%)	352	40 (69.0%)	18 (31.0%)	58	410
*An. coustani*	67 (31.6%)	145 (68.4%)	212	7 (35.0%)	13 (65.0%)	20	232
*An. pharoensis*	16 (32.0%)	34 (68.0%)	50	1 (50.0%)	1 (50%)	2	52
*An. squamous*	6 (66.7%)	3 (33.3%)	9	0 (0.0%)	1 (100%)	1	10
Total	4266 (45.6%)	5094 (54.4%)	9360	1291 (79.3%)	336 (20.7%)	1627 (100%)	10,987
Percentage nighttime vs daytime catches	85.2%			14.8%			100%
*Other mosquito species (Culicines)*							
*Culex quinquefasciatus*	8001 (53.5%)	6960 (46.5%)	14,961	3685 (76.4%)	1139 (23.6%)	4824	19,785
*Mansonia uniformis*	910 (26.6%)	2513 (73.4%)	3423	59 (37.1%)	100 (62.9%)	159	3582
Total	8911 (48.5%)	9473 (51.5%)	17,484 (100%)	3744 (75.1%)	1239 (24.9%)	4983 (100%)	23,367
Percentage nighttime vs daytime catches	77.8%			22.2%			100%

Table 2 shows the results of a generalized linear model fitted using the Bayesian approach to examine the hourly abundance of mosquitoes caught per person between indoor and outdoor locations. The average biting rate was 0.75 for *An. arabiensis* and 0.02 for *An. funestus* indoors, compared to 0.72 and 0.01 outdoors, respectively. The model revealed no significant differences in the indoor versus outdoor biting rates for either species (*An. arabiensis*: relative risk [RR] 0.97, 95% CI 0.93–1.01; *An. funestus*: RR 0.70, 95% CI 0.57–0.85).

**Table 2:** Mean number of mosquitoes per person per hour

Species	Location	Total catch (*n*)	Mean biting rate [95% CI]	RR [95% CI]
*An. arabiensis*	Indoors	5219	0.75 [0.54, 1.03]	1
	Outdoors	5064	0.72 [0.52, 1.00]	0.97 [0.93, 1.01]
*An. funestus*	Indoors	241	0.02 [0.01, 0.04]	1
	Outdoors	169	0.01 [0.006, 0.03]	0.70 [0.57, 0.85]

*CI* Credible interval,* RR* relative risk, *n* number of mosquito catches

### 24-hour biting patterns of the main malaria vectors

Host-seeking activity of the two primary mosquito species, *An. arabiensis* and *An. funestus*, was markedly higher at night than during the daytime. During the nighttime, 8737 *An. arabiensis* and 352 *An. funestus* were collected, while during daytime 1546 *An. arabiensis* and 58 *An. funestus* were collected (Table 3). Overall, daytime biting accounted for 15.03% of *An. arabiensis* catches and 14.15% of *An. funestus* catches. Also, nighttime exposure to *An*. *arabiensis* was greater outdoors (54.5%), while daytime exposure was greater indoors (80.4%). For *An. funestus*, higher exposure was observed indoors, both at nighttime (57.1%) and daytime (69%).

**Table 3:** Mean number of bites per person per hour during daytime and nighttime

Species	Location	Daytime catches			Nighttime catches		
		Total catch (*n*)	Mean [95% CI]	RR [95% CI]	Total catch (*n*)	Mean [95% CI]	RR [95% CI]
*An. arabiensis*	Indoor	1243	0.25 [0.15, 0.43]	1	3976	1.15 [0.84, 1.58]	1
	Outdoor	303	0.06 [0.04, 0.11]	0.24 [0.21, 0.28]	4761	1.38 [1.01, 11.89]	1.20 [1.15, 1.25]
*An. funestus*	Indoor	40	0.004 [0.001, 0.012]	1	201	0.04 [0.02, 0.07]	1
	Outdoor	18	0.002 [0.000, 0.01]	0.44 [0.25, 0.78]	151	0.03 [0.01, 0.05]	0.75 [0.61, 0.93]

*CI* Credible interval,* RR* relative risk, *n* number of mosquito catches

**Fig. 4:**
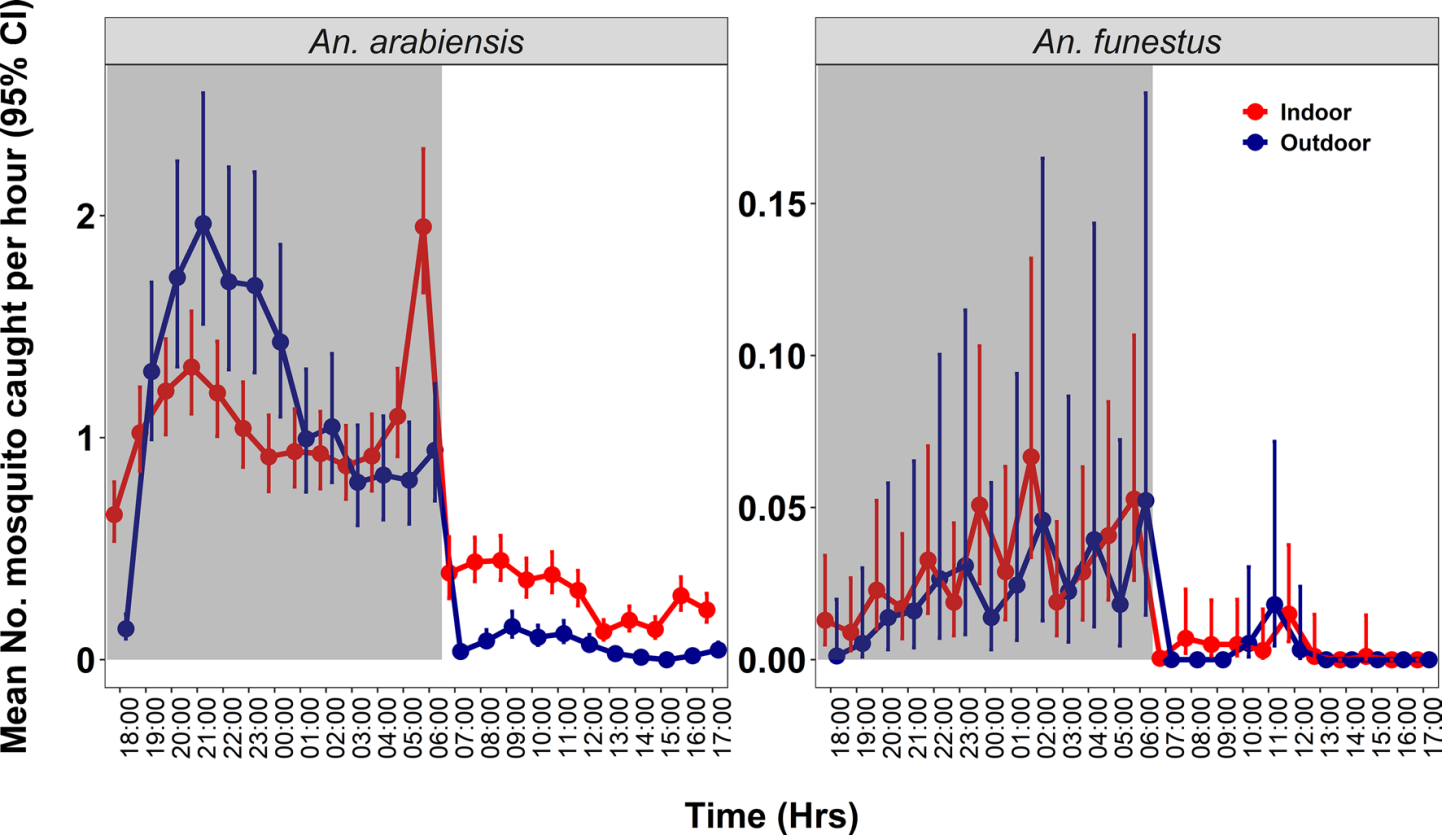
Analysis of the 24-h biting patterns of the main malaria vectors, *Anopheles arabiensis* and *Anopheles funestus* in the study area. CI, Credible interval

During the night, *An. arabiensis* exhibited peak biting activity during the first half of the night between 7 p.m. and 11 p.m., with biting activity starting while significant fractions of people were indoors or outdoors and continuing as people were going indoors and going to bed, then decreasing towards morning. Conversely, *An. funestus* displayed peak indoor biting activity later at night between 1 a.m. and 3 a.m., when most people were asleep and likely under their bed nets. Additional peak activity for both *An. arabiensis* and *An. funestus* was observed during the morning hours both indoors and outdoors, coinciding with the time people were awake, had exited their bed nets and were already outside engaging in routine morning activities. During the rest of the day, *An. arabiensis* showed higher indoor biting rates, with peaks between 7 a.m. and 11 a.m., and again from 4 p.m. onwards, while *An. funestus* had a small indoor biting peak between 10 a.m. and 12 p.m., with a less obvious preference for indoors over outdoors locations (Fig. 4).

The mean number of bites per person per hour during daytime and nighttime shows that *An. arabiensis* had a higher biting rate at night compared to daytime for both the indoor and outdoor locations. Mean indoor nighttime biting activity for *An. arabiensis* was 1.15 bites per person per hour, while outdoor nighttime biting activity was slightly higher at 1.38 bites per person per hour. Daytime biting rates for *An. arabiensis* were relatively lower, with mean indoor biting activity being at 0.25 bites per person per hour and outdoor biting being 0.06 bites per person per hour. *Anopheles funestus* had significantly lower biting rates overall, with mean indoor nighttime biting activity of 0.04 bites per person per hour and mean outdoor nighttime biting activity of 0.03 bites per person per hour. Daytime biting for *An. funestus* was minimal, with indoor rates at 0.004 bites per person per hour and outdoor rates at 0.002 bites per person per hour (Table 3).

### Physiological states, parity and *Plasmodium* infection rates of the malaria vectors collected

**Fig. 5:**
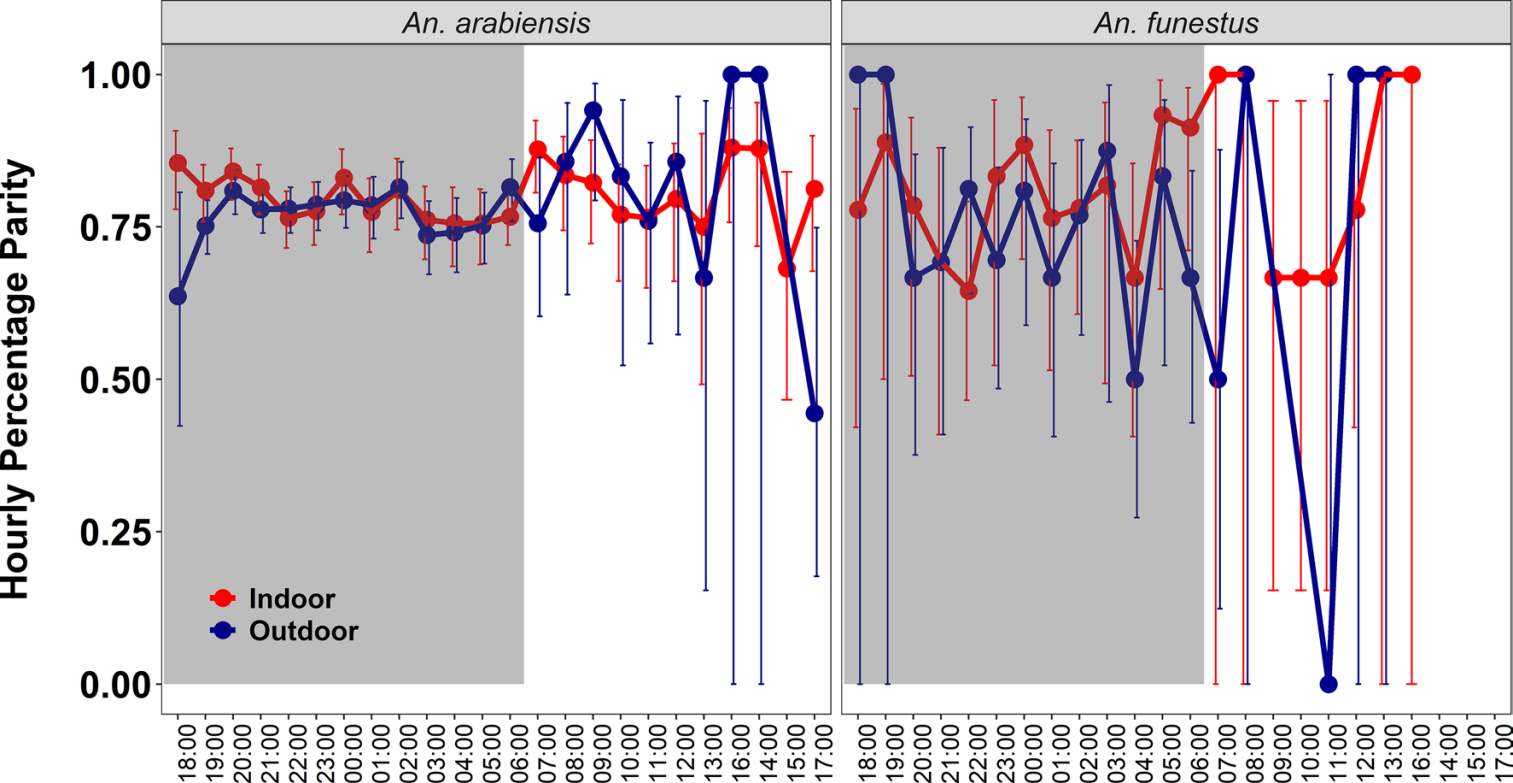
Hourly parity rates in the female *Anopheles arabiensis* and *Anopheles funestus* mosquitoes caught indoors versus outdoors

Of the 10,693 female *An. arabiensis* and *An. funestus* mosquitoes collected, 70.0% were unfed, 21.4% were gravid, 7.0% fed and 1.6% were semi-gravid. Slightly more than half of the host-seeking female *An. arabiensis* (58.4%) and 52.5% of *An. funestus* caught during the daytime were unfed. The parity rate for *An. arabiensis* caught indoors and outdoors during the daytime was 82.57% and 79.47%, respectively, and for *An. funestus* caught indoors and outdoors during the daytime it was 84.44% and 66.67%, respectively. Similarly, during nighttime, the parity rate for *An. arabiensis* caught indoor and outdoors was 78.99% and 78.2%, respectively, and for *An. funestus* caught indoors and outdoors it was 84.67% and 66.86%, respectively. No significant difference was detected in the proportion of parous *An. arabiensis* and *An. funestus* mosquitoes collected indoors (*An. arabiensis*: RR 0.82, 95% CI 0.55, 1.22; *An. funestus*: RR 0.37, 95% CI 0.07, 1.83) and outdoors (*An. arabiensis*: RR 0.95, 95% CI 0.85, 1.07; *An. funestus*: RR 0.37, 95% CI 0.07, 1.83). However, *An. funestus* and *An. arabiensis* caught indoors during daytime and nighttime had higher parity rates compared to the respective outdoor catches between day and night (Table 4). Despite the lower numbers collected during the daytime, the indoor and outdoor hourly parity rates were higher during the daytime than at night (Fig. 5).

**Table 4:** Parity rate indoors versus outdoors during daytime and nighttime

	Location	Daytime catches			No. parous	Nighttime catches	
		No. of parous	Proportion of parous mosquitoes [95% CI]	OR [95% CI]		Proportion parous [95% CI]	OR [95% CI]
*An. arabiensis*	Indoors	635/769	82.57% [79.73, 85.10]	1	2272/2876	78.99% [77.48,80.43]	1
	Outdoors	151/190	79.47% [73.14, 84.63]	0.82 [0.55, 1.22]	3026/3871	78.2% [76.84, 79.43]	0.95 [0.85, 1.07]
*An. funestus*	Indoors	38/45	84.44% [70.80, 92.40]	1	127/150	84.67% [70.79,92.40]	1
	Outdoors	6/9	66.67% [33.34, 88.89]	0.37 [0.07, 1.83]	113/169	66.86% [33.34,88.89]	0.37 [0.07, 1.83]

*CI* Credible interval,* OR* odds ratio

Regarding *Plasmodium* sporozoite infections, a total of 3095 female *An. arabiensis* mosquitoes (1832 collected indoors and 1263 outdoors) were tested, with only one sporozoite-positive mosquito found in each subset. The one *Plasmodium*-positive mosquito caught indoors was captured between 7:00 a.m. and 7:50 a.m., while the one *Plasmodium*-positive mosquito caught outdoors was captured between 11:00 p.m. and 11:50 p.m. Additionally, 178 female *An. funestus* mosquitoes (97 indoors and 81 outdoors) were tested out of 410 collected during the entire study period, but no infections were detected.

### Human exposure to mosquito bites indoors and outdoors

**Fig. 6:**
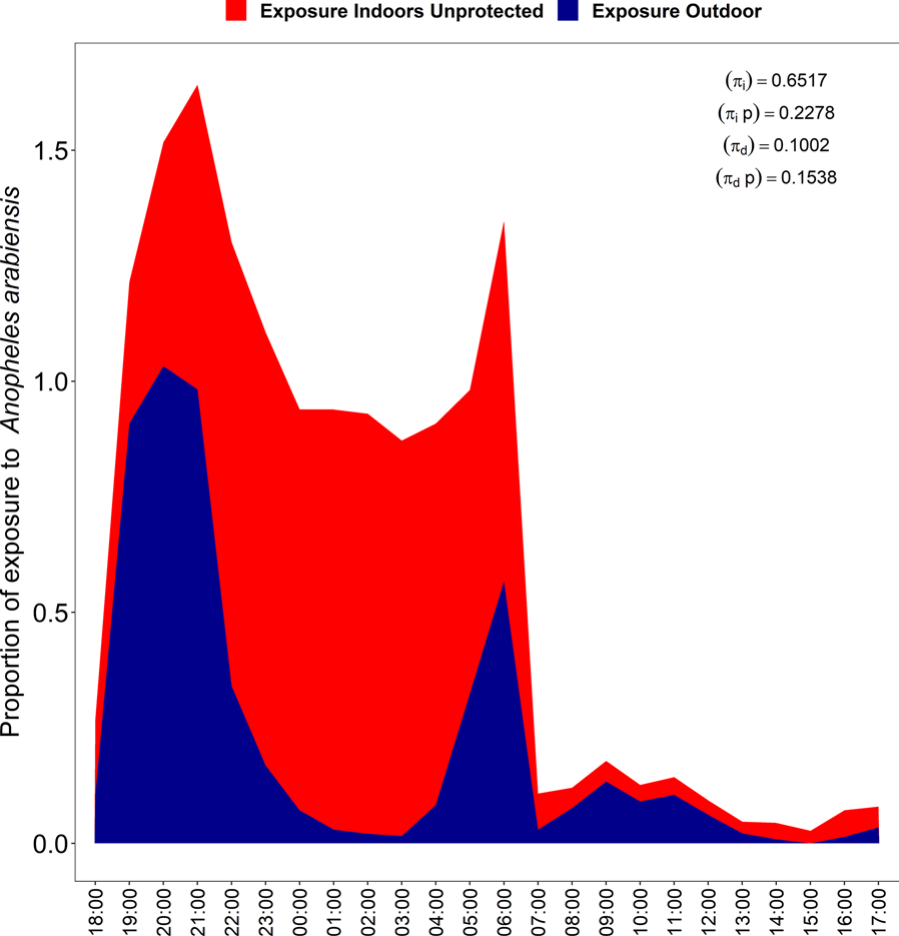
Proportions of exposure to *Anopheles arabiensis* bites both indoors and outdoors

**Fig. 7:**
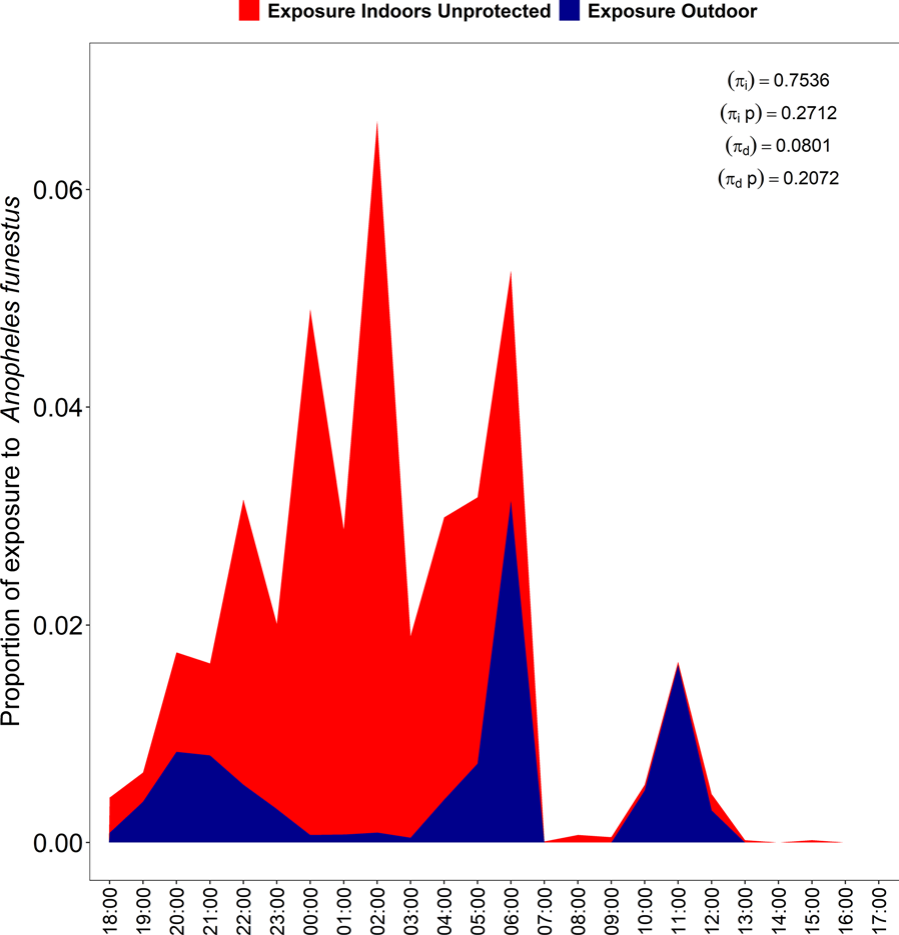
Proportions of exposure to *Anopheles funestus* bites both indoors and outdoor

Analysis of the human behavior data alongside the entomological data showed that overall, the majority of exposures to *An. arabiensis* occurred outdoors during the first part of the night between 7 p.m. and 11 p.m., before most people went to bed, with additional exposure in the morning hours between 4 a.m. and 6 a.m. when most people were awake. Outdoor exposure continued throughout daytime hours when most people were active outdoors, with a small peak between 8 a.m. and 10 a.m. (Fig. 6). In contrast, the bulk of exposure to *An. funestus* occurred indoors during the late-night hours between 1 a.m. and 2 a.m. when most people were indoors and under bed nets. Outdoor exposure to *An. funestus* was slightly higher during the first half of the night between 8 p.m. and 10 p.m. when people were still outdoors, and during early morning hours between 4 a.m. and 6 a.m. During the daytime, exposure to *An. funestus* occurred mostly indoors across most of the hours except during midday when outdoor exposure increased coincidentally with the biting pattern, peaking from 10 a.m. to 12 p.m. (Fig. 7).

When accounting for location, the proportion of exposure to *An. arabiensis* and *An. funestus* bites occurring indoors (*πi*) for unprotected individuals was 0.652 and 0.754, respectively, while for protected individuals or users of long-lasting insecticidal nets (LLINs) (*πi*, *p*), the proportion of exposure occurring indoors for LLIN users (*πi*, *p*) was 0.229 and 0.271 respectively. When accounting for time, the proportion of exposure to *An. arabiensis* and *An. funestus* bites occurring in daylight for unprotected individuals was (*πd*) was 0.100 and 0.080, respectively, while for LLIN users, the proportion of exposure occurring during daylight (*πd*, *p*) was 0.154 and 0.207, respectively. For children aged < 5 years, the proportion of exposure occurring indoors (*πi*) was 0.689 for *An. arabiensis* and 0.815 for *An. funestus* while the proportion of exposure occurring indoors for those protected by LLINs (*πi*, *p*) was 0.239 and 0.337 respectively. In terms of time, the proportion of exposure occurring during daylight (*πd*) was 0.097 for *An. arabiensis* and 0.082 for *An. funestus*, while the proportion of exposure occurring during daylight for those protected by LLINs (*πd*, *p*) was 0.164 and 0.249 respectively (Table 5).

**Table 5:** Mosquito–human behavior interactions

Variable	Exposure^a^	Proportion of exposure to:	
		*Anopheles arabiensis*	*Anopheles funestus*
Exposure of all family members	Indoor exposure risk for individuals not using ITNs (*πi*)	0.65	0.75
	Indoor exposure risk for individuals using ITNs (*πi*, *p*)	0.23	0.27
	Daytime exposure risk for individuals not using ITNs (*πd*)	0.10	0.08
	Daytime exposure risk for individuals using ITNs (*πd*, *p*)	0.15	0.21
Exposure of children below school age	Indoor exposure risk for individuals not using ITN (*πi*)	0.69	0.82
	Indoor exposure risk for individuals using ITN (*πi*, *p*)	0.24	0.34
	Daytime exposure risk for individuals not using ITNs (*πd*)	0.10	0.08
	Daytime exposure risk for individuals using ITNs (*πd*, *p*)	0.16	0.24
Exposure of females	Indoor exposure risk for individuals not using ITN (*πi*)	0.51	0.41
	Indoor exposure risk for individuals using ITN (*πi*, *p*)	0.23	0.13
	Daytime exposure risk for individuals not using ITNs (*πd*)	0.17	0.10
	Daytime exposure risk for individuals using ITNs (*πd*, *p*)	0.16	0.12
Exposure of males	Indoor exposure risk for individuals not using ITN (*πi*)	0.52	0.41
	Indoor exposure risk for individuals using ITN (*πi*, *p*)	0.23	0.13
	Daytime exposure risk for individuals not using ITNs (*πd*)	0.16	0.10
	Daytime exposure risk for individuals using ITNs (*πd*, *p*)	0.16	0.12

*﻿a: ITNs* Insecticide-treated nets

### Routine household activities within the peri-domestic area

**Fig. 8:**
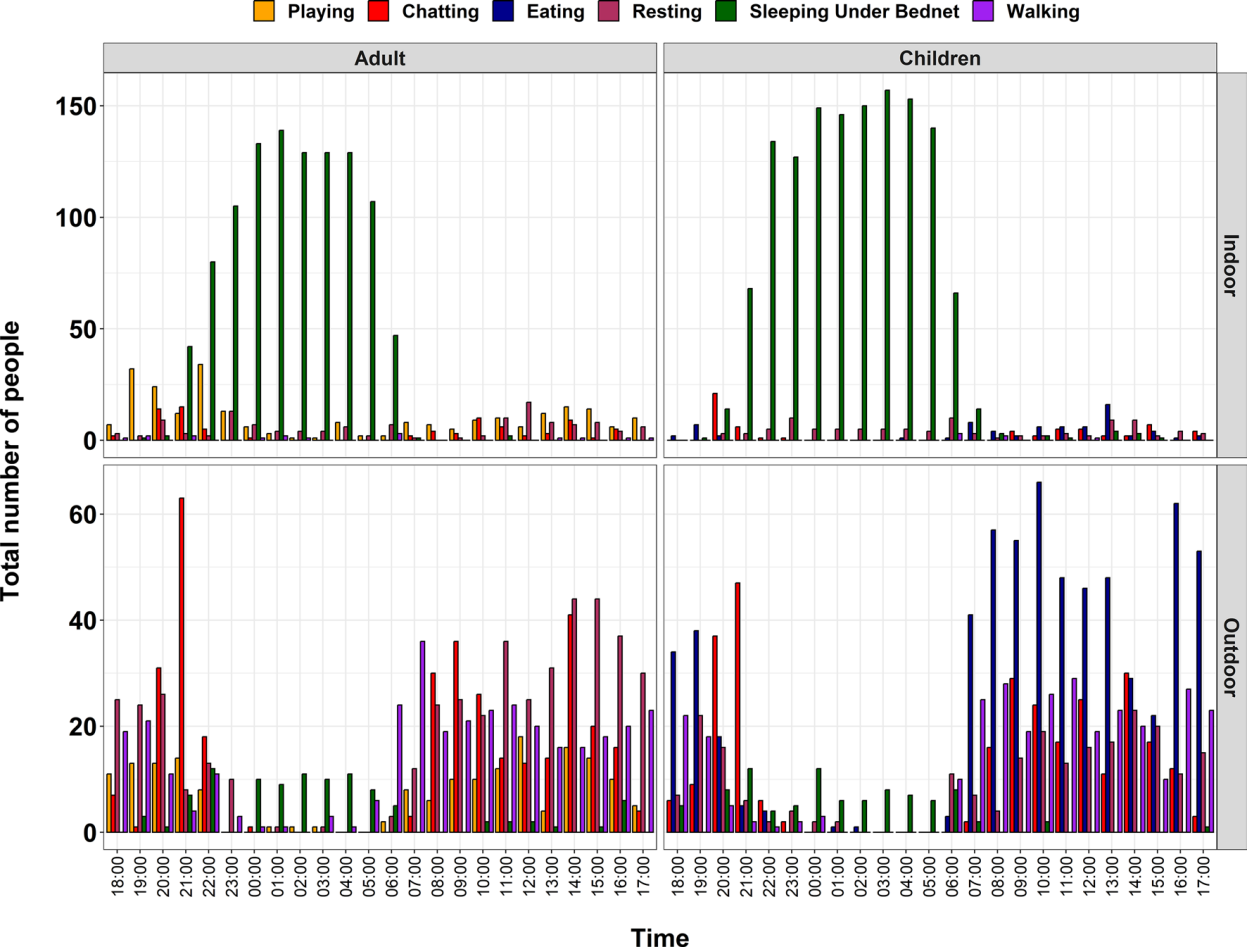
Common household activities exposing family members to mosquito bite

During early night hours (6 p.m. to 10 p.m.), most family members were indoors but outside of their bed nets, mainly resting, chatting outside bed nets, playing and walking around, and some were still outdoors engaged in almost the same activities as just mentioned. From 10 p.m. to 4 a.m., most people (adults and children) were indoors and under the protection of bed nets. Between 4 a.m. and 7 a.m. the proportion of people outdoors started to increase, and during broad daylight between 7 a.m. and 5 p.m. significantly more people were present outdoors, walking around, eating, resting under tree shades or verandas and playing. Popular activities that kept people indoors during the daytime included chatting outside bed nets, resting after work, playing, walking around and eating (Fig. 8).

### Results of the community survey: sociodemographics of study participants

Of the 182 household representatives interviewed, 72.0% (*n* = 131) were females and 28.0% (*n* = 51) were males (Table 6). The largest age group was the 25–34 year-old age group, accounting for (48.9% (*n* = 89) of the participants. Approximately 67.0% (*n* = 122) of participants had completed primary education. The primary economic activity was crop production, involving 78.6% (*n* = 143) of the participants, with 48.9% (*n* = 89) having a minimum monthly income of at least 200,000 Tanzanian shillings. More than half of households had four to six members (58.2%, *n* = 106) and > 42.9% (*n* = 78) of households had at least two children; pregnant women were present in 10.4% (*n* = 19) of households. Regarding malaria prevention, 57.1% (*n* = 104) of households had at least one to two bed nets, and 39.0% (*n* = 71) had three to four occupants sleeping under bed nets. Most houses were typically constructed with mud and grass thatched roofs (33.5%, *n* = 61), open eaves (77.5%, *n* = 141) and unscreened windows (73.6%, *n* = 134), while 46.2% (*n* = 84) of houses had holes in the walls (Table 7).

**Table 6:** Sociodemographic characteristics of study participants

Variables	Overall values ( N= 182 persons interviewed)
*Sex of respondent*	
Female	131 (72.0%)
Male	51 (28.0%)
*Age of household members (years)*	
18–24	10 (5.5%)
25–34	89 (48.9%)
35–44	34 (18.7%)
45–54	24 (12.6%)
55–64	18 (9.9%)
65+	8 (4.4%)
*Educational status of household members*	
Primary/lower	122 (67.0%)
Secondary	44 (24.2%)
University/college	16 (8.8%)
*Occupation of the respondent*	
Retail business	9 (4.9%)
Crop production and livestock-keeping	16 (8.8%)
Crop production	143 (78.6%)
Livestock keeping	11 (6.0%)
Others	3 (1.6%)
*Monthly income (Tanzanian shillings)*	
100,000	26 (14.3%)
200,000	89 (48.9%)
300,000+	67 (36.8%)
*Family size*	
1–3	46 (25.3%)
4–6	106 (58.2%)
6+	30 (16.5%)
*Number of children in the household aged < 5 years*	
0	19 (10.4%)
1–2	78 (42.9%)
3–4	555(30.2%)
5+	30 (16.5%)
*Pregnant women present*	
Yes	19 (10.4%)
No	163 (89.6%)

Values in table are presented as a count (*n*) with the percentage in parentheses

**Table 7:** House characteristics and interventions used

Variables	Overall (*N* = 182 persons interviewed)
*Number of bed nets present*	
1–2	104 (57.1%)
3–4	54 (29.7%)
5+	24 (13.2%)
*Number of people sleeping under a bed net*	
0	27 (14.8%)
1–2	51 (28.0%)
3–4	71 (39.0%)
5+	33 (18.1%)
*Window status*	
Screened	48 (26.4%)
Unscreened	134 (73.6%)
*House type*	
Plastered brick walls + metal sheet roof	2 (13.2%)
Unplastered brick walls + metal sheet roof	21 (11.5%)
Mud wall + metal sheet roof	33 (18.1%)
Plastered brick walls + grass thatched roof	8 (4.4%)
Unplastered brick walls + grass thatched roof	35 (19.2%)
Mud wall + grass thatched roof	61 (33.5%)
*Door status*	
Closed	121 (65.4%)
Open	64 (34.8%)
*Eave status*	
Closed	41 (22.5%)
Open	141 (77.5%)
*Wall status*	
Holes absent	98 (54.8%)
Holes present	84 (46.2%)

Values in table are presented as a count (*n*) with the percentage in parentheses

### Knowledge and perceptions about day-biting mosquitoes

Regarding awareness of day-biting mosquitoes, 135 (74.2%) of the representatives interviewed said they were aware of mosquitoes biting during the daytime and 94.8% (*n* = 128) of them admitted experiencing mosquito bites during the daytime. Interestingly, only 28.9% (*n* = 39) of those interviewed knew that malaria could be transmitted by day-biting mosquitoes, while the majority 57.8% (*n* = 78) believed that day-biting mosquitoes were just common nuisance biters. Only 26.7% (*n* = 36) of those interviewed reported making any deliberate efforts to guard against day-biting mosquitoes. The bulk of biting was reported to occur during the early morning hours between 7 a.m. and 9 a.m. (33.3%, *n* = 45) and during the late morning hours between 9 a.m. and 11 a.m. (23.7%, *n* = 32). Children aged < 5 years (28.9%, *n* = 39), mothers (25.9%, *n* = 35) and school children (20.7%, *n* = 28) were identified as the most at-risk groups, primarily because most of these people spent much of their daytime at home. Activities that kept most of them indoors during the daytime were sleeping (28.1%, *n* = 38), house chores (15.6%, *n* = 21) and resting after work (26.7%, *n* = 36), while activities like resting in the shade of trees (5.9%, *n* = 35), cooking (19.3%, *n* = 26), eating (25.2%, *n* = 34) and sharing stories kept them outdoors (Table 8).

**Table 8:** Knowledge and perceptions about day-biting mosquitoes

Variables	Percentages
*Aware of day-biting mosquitoes (N = 182)*	
Yes	135 (74.2%)
No	35 (19.2%)
I don’t know	12 (6.6%)
*Experienced mosquito bites during daytime (N = 135)*	
Yes	128 (94.8%)
No	6 (4.4%)
I don’t know	1 (0.4%)
*Aware if malaria can be transmitted by day-biting mosquitoes (N = 135)*	
Yes	39 (28.9%)
No	78 (57.8%)
I don’t know	18(13.3%)
*Time of day mosquitoes bite (N = 135)*	
Early morning between 7 a.m. and 9 a.m.	45 (33.3%)
Late mornings between 9 a.m. and 11 a.m.	32(23.7%)
Midday between 11 a.m. and 2 p.m.	17 (12.6%)
Early evening between 2 p.m. and 4 p.m.	13 (9.6%)
Late evening between 4 p.m. and 7 p.m.	20 (14.8%)
I don’t know	8 (5.9%)
*Make efforts to guard against day biting mosquitoes (N = 135*)	
Yes	36 (26.7%)
No	90 (66.7%)
I don’t know	9 (6.7%)
*Family members always present at home during the daytime (N = 135)*	
Children aged ≤ 5 years	39 (28.9%)
School-aged children	28 (20.7%)
Elderly (65+ years and/or disabled	7(5.2%)
Fathers	14 (10.4%)
Mothers	35 (25.9%)
*Reasons for staying indoors (N = 135)*	
Sickness	11 (8.1%)
House chores	21 (15.6%)
Sleeping	38 (28.1)
Reading	7 (5.2%)
Resting after work	36 (26.7%)
Eating	9 (6.7%)
Other	3 (2.2%)
*Reasons for staying outdoors (N = 135)*	
Extreme heat inside	11 (8.1%)
Cooking	26 (19.3%)
Sleeping	15 (11.1%)
Eating	34 (25.2%)
Resting in the shade of trees	35 (25.9%)
Sharing stories	9 (6.7%)
Other	5 (3.7%)

### A day in the respondent’s life during wet and dry seasons

During the wet season, nearly half (49.6%, *n* = 67) of the respondents reported waking up between 5 a.m. and 6 a.m., and 41.5% (*n* = 56) reported waking up between 6 a.m. and 7 a.m. Major indoor activities as reasons for waking up early were house chores (60.7%, *n* = 82) while outdoor activities included cooking breakfast (28.2%, *n* = 38) and cleaning the compound (31.1%, *n* = 42). Farming activities dominated the morning activities away from the peri-domestic spaces (81.5%, *n* = 110). Similarly, during the dry season, most of the respondents (46.7%, *n* = 63) reported waking up between 6 a.m. and 7 a.m, with the major indoor activities cited for rising being cooking breakfast (60.7%, *n* = 82) and cleaning the compound (37.0%, *n* = 50) and the major outdoor activities as cooking breakfast (24.0%, *n* = 32) and cleaning the compound (31.1%, *n* = 42).

## Discussion

Current malaria prevention tools, notably ITNs and IRS, have yielded significant gains but remain challenged by numerous biological threats such as insecticide resistance and variations in vector behaviors [33, 64–68]. These interventions primarily target the nocturnal and indoor-biting behaviors of the major Afrotropical malaria vectors, including *An. gambiae* s.l. and *An. funestus*, which are the main vectors of malaria in Tanzania. These vectors are most active during the night when people are typically protected by ITNs. However, there is mounting evidence showing significant extended biting activity among these vectors that coincides with periods when people are engaged in activities such as farming, fetching water and other livelihood tasks, i.e. when they are unprotected by ITNs [25, 42, 69–71]. One reason for the historical neglect of these biting patterns is the biases in traditional entomological survey methods, which typically overlook daytime mosquito activity [72, 73]. Since such atypical behaviors might reflect a broader risk spectrum and multiple potential protection gaps beyond the reach of ITNs, there is a need to investigate these patterns in different contexts. This study therefore investigated the 24-h patterns of mosquito bites and human exposures, focusing on both the diurnal and nocturnal biting patterns of the malaria vectors in rural southeastern Tanzania.

Overall, the findings of this study suggest that while most of the biting activity of the two dominant malaria vectors, *An. funestus* and *An. arabiensis*, remains at night, the biting risk posed by day-biting malaria vectors is not insignificant. This new paradigm challenges the current near-universal focus on nighttime interventions and underscores the need to address residual malaria transmission through enhanced vector control strategies that consider both diurnal and nocturnal biting patterns.

We observed that nocturnal biting by *An. arabiensis* peaked between 7 p.m. and 11 p.m., while *An. funestus* exhibited a delayed peak, being most active from 1 a.m. to 3 a.m.. These results are inconsistent with those reported in a study in Kamuli district, Uganda where the majority of the biting by *An. gambiae* s.l. and *An. funestus* group occurred between 11 p.m. and 5 a.m., a period when most people are typically under ITNs [74]. However, these biting patterns observed in the present study have also been observed in multiple previous studies [25, 75–78], and *An. arabiensis* mosquitoes, in particular, are known to be very active in the early evening and early night hours, often readily biting outdoors or indoors. This species is therefore less readily impacted by ITNs than the more endophilic, endophagic and late-biting *An. funestus* [45, 79]. The daytime host-seeking collections, from 6 a.m. to 7 p.m., accounted for 14–15% of the total host-seeking mosquitoes for both species. The findings showed, however, that the daytime hourly pattern of the host-seeking females was only marginally different between these species, with the observed differences being mostly due to the higher densities of *An. funestus* caught in this study. Notably, *An. arabiensis* displayed increased activity from 7 a.m. to 11 a.m. and a sharp rise in activity the early evening from 6 p.m. to 7 p.m., whereas *An. funestus* showed smaller daytime peaks from 10 a.m. to 12 p.m. This daytime activity of major malaria vectors, though modest, aligns with human activities, both indoors and outdoors, such as household chores, farming and fetching water, during which people are unprotected by ITNs. The findings are a piece of additional evidence to several recent findings of extended biting by malaria vectors in Africa [23, 35, 80]. In particular, they confirm the now seemingly ubiquitous patterns of *An. funestus*, the predominant vector in our study area, having extended morning to mid-morning biting activity [34, 35, 37, 81].

Analysis of the indoor and outdoor biting rates also revealed significant differences between mosquito species and between the nocturnal and diurnal time ranges. At night, just over half (54.5%) of *An. arabiensis* were caught outdoors, but during the day this capture rate increased significantly to 80.4% indoors. Similarly, *An. funestus* mosquitoes were primarily caught indoors both at night (57.1%) and during the day (69%). The greater percentages of indoor biting during the day are likely driven by temperature differences, with indoor areas being cooler than outdoor areas during the day. More importantly, these patterns suggest that while ITNs are effective in targeting indoor-biting mosquitoes at night [74], additional tools or approaches are needed to cover the fraction of biting that happens during the daytime indoors. The other main vector control tool, IRS, is likely to continue being effective during both the day and night [31], but most people do not use any personal protection against malaria vectors during the day. Moreover, the effectiveness of ITNs is absent during the day except in cases where the vectors remain fully susceptible, where community benefits arising from the mass mosquitocidal effects of ITNs might be more impactful [8, 10].

Another parameter examined in our was the physiological states and parity rates of the mosquitoes collected. A high proportion of unfed mosquitoes were found during the daytime, with > 58% of day-biting and 72% of diurnal-biting mosquitoes being unfed. These values likely reflect the host-seeking state at which the mosquitoes were collected during their diurnal and nocturnal activity cycles. The parity rates were generally high but they were notably higher in the daytime catches for *An. arabiensis* (82.57% indoors and 79.47% outdoors) compared to the nighttime catches. For *An. funestus*, the parity rates were comparable between the daytime and nighttime catches. These high parity rates likely reflect the near absence of newly emerged unfed mosquitoes foraging indoors at these hours. More importantly, they suggest that many of these mosquitoes potentially may have been exposed to infective blood meals and survived more than one gonotrophic cycle, thereby increasing the risk of malaria transmission. Indeed, higher parity rates are regularly reported for *An. funestus* compared to *An. arabiensis* [23, 56–58], and in households far from aquatic habitats [82], but are more likely to be the result of our biased sampling design, which focused mostly in and around households. Additionally, *Plasmodium*-positive mosquitoes were detected in both daytime and nighttime collections of *An. arabiensis*. These results imply that there is indeed a risk of malaria infections associated with these day-biting mosquitoes, irrespective of the small numbers collected during daytime; as such, they are in agreement with the results of a recent study from the Central African Republic [23].

Human behaviors and activities significantly contribute to exposure risk during different times of the day and night [25, 42, 83]. In the present study, several activities, including farming, fetching water and other tasks associated with daily livelihood-associated tasks, were identified as having the potential to elevate the risk of mosquito bites during periods of high mosquito activity when people are not protected by ITNs. These findings correlate with previous observations in various settings, including East Africa [25, 42, 84], West Africa [63, 64], several other African settings [36, 71, 85] and the South Pacific [86]. Our findings showed overlaps between human activities and mosquitoes both indoors and outdoors, in the mornings and evenings. This overlap may contribute to significant human-vector contacts [71, 76, 87, 88, 89]. The authors of previous studies reported that in the evenings people commonly perform multiple peri-domestic activities before eventually going under their nets. These behaviors typically result in lower protective efficacy of ITNs, even in settings where the ownership and use of ITNs are high [7, 36, 83, 90]. Unfortunately, community surveys also revealed a general lack of awareness of the risk posed by day-biting mosquitoes, which has implications for malaria prevention practices. Educating communities about the importance of protection during daytime activities and implementing strategies that extend protection beyond nighttime, such as daytime repellents or protective clothing, could therefore be critical in reducing malaria transmission.

The findings of this study highlight the limitations of current malaria control interventions that primarily target nocturnal and indoor-biting mosquitoes. The modest but significant daytime and outdoor biting activity observed in our study necessitates a re-evaluation of vector control strategies to include measures that address mosquito activity throughout the entire 24-h period. Compared to the 14.8% *Anopheles* biting observed in this study, earlier studies demonstrated that these fractions could reach 20–30% in some settings [23], further emphasizing the need to expand both the surveillance and control programs to include 24-h cycles. Future research should investigate the full implications of this extended spectrum of biting on malaria risk in the villages and whether significant additional interventions are warranted. One strategy which can be used to address this is the careful re-evaluation of existing vector control tools. For example, while ITNs may be less effective on day-biting mosquitoes, IRS, by targeting resting mosquitoes at all times, can remain an effective control method regardless of the mosquito biting patterns [91–93].

Indeed, a careful evaluation of current interventions is essential for safeguarding at-risk populations and achieving the goal of malaria elimination, especially in regions with persistent transmission despite high ITN coverage. Integrating comprehensive entomological and human behavior data can inform more effective and responsive malaria control measures, ultimately enhancing the effectiveness of interventions and reducing the burden of malaria in endemic areas [25, 42, 83, 94]. Interventions such as larval source management, which targets mosquitoes at their source could be highly effective as a complementary tool alongside ITNs and IRS [28]. Personal protection, such as repellents or long-sleeved clothing and mosquito repellents, are other options, although their consistent use in low-income settings might be low. Regarding ITNs, even though these are typically used at night, there is scope for ITNs or even untreated nets for young children and babies sleeping indoors during the day, as well as for any invalid person or elderly person who spends most of their time indoors. Overall, the extended range of locations and times when biting exposure occurs highlights the need for interventions that can protect individuals both indoors and outdoors throughout the day, particularly in areas with high malaria transmission where outdoor activities are common.

The study had several limitations that could potentially bias our findings. Firstly, it was conducted during the dry season, when adult *Anopheles* mosquito populations are typically low; we could not estimate 24-h patterns and malaria transmission risks during the wet season due to time and funding constraints. Moreover, in the dry season, people are more likely to spend time outdoors compared to the rainy season, which may likely have led to an overestimation of exposure to risk outdoors or underestimated exposure risk indoors Secondly, while molecular analysis was performed, it relied heavily on findings from previous studies in the region due to limited time and laboratory resources. Thirdly, for confidentiality, human behavior observations in the peri-domestic area were conducted in a subset of households with a literate volunteer, potentially missing observations in other households or when volunteers were unavailable. Finally, the study was limited to two villages, which may not be representative of the entire population or environment, restricting the generalizability of our findings.

We recommend comprehensive longitudinal studies with larger sample sizes to fully understand the role of day-biting in sustaining persistent malaria transmission and its implications for current vector control tools. Additionally, with increasing evidence of day-biting mosquitoes, we recommend including diurnal biting mosquitoes in routine entomological surveys to accurately estimate their contribution to persistent malaria transmission and inform the development and deployment of effective vector control interventions.

## Conclusions

This study provides important updates to our understanding of the biting patterns of the main malaria vectors, *An. arabiensis* and *An. funestus*, in rural southeastern Tanzania, highlighting substantial biting activity outside the protection of ITNs. While nocturnal biting remains predominant, with peaks from 7 p.m. to 11 p.m. for *An. arabiensis* and from 1 a.m. to 3 a.m. for *An. funestus*, this study has revealed considerable daytime biting activity, especially in the mornings and early evenings, coinciding with human activities. This broader biting spectrum suggests that current vector control strategies, which primarily target nocturnal behaviors, may be insufficient. The higher parity rates observed during daytime collections indicate the potential for significant malaria transmission risk during the day when people are not under ITNs. Therefore, complementary strategies are needed to holistically suppress vectors regardless of biting patterns, such as using larval source management, or to extend personal protection against mosquitoes active during these times, such as by using repellents. Additionally, intensive health education and community engagement are crucial to raise awareness of the risks associated with daytime mosquito activity and to promote protective measures, ultimately contributing to more effective malaria control and elimination efforts. Finally, further studies are also required to better understand the extent of this extended biting and its implications for disease transmission and control.

## Data Availability

All data supporting the main conclusions of this article are included within the article and its additional files.

## Abbreviations

DN-Mini: Miniaturized double net trap
HBOs: Human behavior observations
IRS: Indoor residual spraying
ITNs: Insecticide-treated nets
